# Factors influencing prescribing behaviour of physicians in Greece and Cyprus: results from a questionnaire based survey

**DOI:** 10.1186/1472-6963-9-150

**Published:** 2009-08-20

**Authors:** Mamas Theodorou, Vasiliki Tsiantou, Andreas Pavlakis, Nikos Maniadakis, Vasilis Fragoulakis, Elpida Pavi, John Kyriopoulos

**Affiliations:** 1 Department of Health Economics & Management, Open University of Cyprus, Cyprus; 2 Department of Health Economics, National School of Public Health, Athens, Greece; 3 Department of Health Services Organisation & Management, National School of Public Health, 196 Alexandras Avenue, 115 22 Athens, Greece

## Abstract

**Background:**

Over the past few decades, drug and overall healthcare expenditure have risen rapidly in most countries. The present study investigates the attitudes and the factors which influence physician prescribing decisions and practice in Greece and Cyprus.

**Methods:**

A postal questionnaire was developed by researchers at the Department of Health Economics at the National School of Public Health in Greece, specifically for the purposes of the study. This was then administered to a sample of 1,463 physicians in Greece and 240 physicians in Cyprus, stratified by sex, specialty and geographic region.

**Results:**

The response rate was 82.3% in Greece and 80.4% in Cyprus. There were similarities but also many differences between the countries. Clinical effectiveness is the most important factor considered in drug prescription choice in both countries. Greek physicians were significantly more likely to take additional criteria under consideration, such as the drug form and recommended daily dose and the individual patient preferences. The list of main sources of information for physicians includes: peer-reviewed medical journals, medical textbooks, proceedings of conferences and pharmaceutical sales representatives. Only half of prescribers considered the cost carried by their patients. The majority of doctors in both countries agreed that the effectiveness, safety and efficacy of generic drugs may not be excellent but it is acceptable. However, only Cypriot physicians actually prescribe them. Physicians believe that new drugs are not always better and their higher prices are not necessarily justified. Finally, doctors get information regarding adverse drug reactions primarily from the National Organisation for Medicines. However, it is notable that the majority of them do not inform the authorities on such reactions.

**Conclusion:**

The present study highlights the attitudes and the factors influencing physician behaviour in the two countries and may be used for developing policies to improve their choices and hence to increase clinical and economic effectiveness and efficiency.

## Background

Over the past few decades, pharmaceutical expenditure has risen rapidly in most western countries and this has been a reason for concern to policymakers, who have reacted with healthcare reforms and measures to guarantee the sustainability of their health care systems [1]. In the countries of the Organisation for Economic Cooperation and Development (OECD), pharmaceutical expenditure accounts, on average, for about 1.5% of the Gross Domestic Product (GDP). It is notable however that it grows, in real terms, by 4.6% annually, which is higher than the growth rate of total healthcare expenditure or the growth rate of GDP [2]. Alongside the concern regarding the growth of pharmaceutical expenditure, there is also a increasing concern regarding irrational, inappropriate, or sometimes even harmful prescribing [3,4]. The latter matter has two manifestations. On the one hand, there is plenty of evidence from observational or experimental studies that, for several reasons many of which have been identified, eligible patients are not always prescribed the pharmaceutical therapies indicated for their condition. On the other hand, there is also evidence about over and misuse of pharmaceutical products. The consequence of the above can be the loss of health and quality of life benefit for patients and society and the increase of health care expenditure [5]. Thus, for health and economic reasons, it is important to follow the recommended optimal and established drug prescription guidelines.

In this context, a lot of research is trying to analyse and to understand the factors which influence physician prescribing decisions and practice. The related literature suggests several factors that may have a role in influencing the prescribing behaviour of physicians [6-9]. Some factors are fixed and they do not offer any opportunity for modification and improvements in prescribing behaviour. Such factors for instance include, the age and sex of the physician or the patient, the socio-economic characteristics of the practicing area or the reimbursement status of therapy [10,11]. On the other hand, there are factors which can be influenced and in turn cause a modification to the prescribing behaviour of physicians. Such factors may be the under and post graduate education and the experience of the physician, various social factors, the number of practitioners in a practice and others [12-15].

It is notable that no other study has attempted so far to analyse the prescribing behaviour and its determinants amongst Greek or Cypriot primary care physicians. Therefore, we carried out a survey in order to investigate the prescribing attitudes of physicians in these two countries and in the present paper we present the main survey results. The paper outlines in a comparative and detailed way the main factors influencing the decision making and the drug prescription choices of physicians in the two countries. More specifically, it reveals the criteria which justify prescription choice, the sources of physician information, the attitudes towards generic or new innovative drugs, the importance of the drug cost in the decision, etcetera. This information can help policy makers to identify the measures needed to improve the effectiveness of health policy and consequently it can contribute towards a greater economic and clinical efficiency and effectiveness in the two countries under consideration.

### Organisation of Health System in Greece and in Cyprus

The Greek health care system has characteristics from both the Beveridge (Social Security) and the Bismarck (Social Insurance) health organisation model. Specifically, a National Health Service (NHS) was established in 1983, with the aim to provide on behalf of the state health care services to all citizens. In this context, services are provided by public secondary and tertiary care hospitals and by primary care units, which are located in semi-urban and rural areas. There are also a few thousand primary care physicians doing after graduation compulsory training in rural areas. The NHS is funded by taxation and by payments coming from many existing Public Insurance Sickness Funds. However, it is notable that the Sickness Funds also operate on their own several primary care health units and hospitals. Finally, alongside the above there is a significant in size private health care sector, comprising freelancing or contracted physicians, diagnostic centres and hospitals. There are no general practitioners, to act as gatekeepers to the healthcare system in Greece. Thus, regarding their health problem, patients can consult with any physician within the primary or secondary health care system. In Cyprus healthcare delivery depends on both public and private health units as well. The public sector is mostly responsible for the provision of secondary and tertiary care and the private sector is responsible for the provision of primary health care services. It is notable that in this country as well there is no gate-keeping system at present, meaning that patients are free to select and consult with any physician of their choice [16].

## Methods

A questionnaire was developed by the Department of Health Economics at the National School of Public Health in Greece, specifically for the purposes of the survey. The questionnaire is divided into seven different sections: the first is designed to investigate the determinants of physician prescribing behaviour and their main sources of information; the second reflects their opinion about the cost of pharmaceuticals to the patient; the third section focuses on attitudes towards the prescription of generics; the fourth section reflects attitudes towards new pharmaceutical products; the fifth section is about adverse drug reactions and safety; the sixth section focuses on pharmaceutical company sales representatives; and the last section includes questions about the demographic characteristics of the person answering. In total, the questionnaire included 47 semi-closed questions. It was piloted to a group of 217 physicians in Greece in the period between the November of 2006 and January of 2007. The physicians who participated in the pilot study made significant comments towards the improvement of the instrument and all of their recommendations were taken into consideration and were incorporated in the final questionnaire.

The proportional stratified sampling technique was used to draw a sample, on the basis of physician geographical region, specialty and sex. Excluded from the sample were physicians who were not authorised to prescribe, either because they were still interns or because they belonged to a specialty that is not permitted to prescribe (radiology, nuclear medicine, microbiology, haematology, anaesthesiology, forensic medicine). In Greece, a sample of 1,463 physicians was randomly selected for the purpose of the study and in Cyprus the questionnaire was sent to 240 physicians also randomly selected in a similar manner. In both countries, the final questionnaire, a cover letter and a prepaid return envelope were mailed to the physicians from the 1^st ^of April 2007 to the 30^th ^of May 2007. The study is non interventional and it does not involve patients and hence no ethical approval was needed. Nonetheless, it was undertaking according to the ethical standards and procedures set for this type of research in both countries and academic institutions involved. Data collection followed by quality control, codification, recording and statistical analysis with SPSS (ver15.0).

## Results

### Demographic characteristics

1,204 physicians from Greece participated in the study (response rate: 82.3%) and 193 in Cyprus (response rate: 80.4%). In Greece 79.9% were male and 20.1% female, whilst in Cyprus the percentages were 69.8% and 30.2%, respectively. As indicated in Table 1, almost half of the participants in Cyprus (46.8%) were between 41–50 years of age, whilst in Greece the largest proportion of participants (35.9%) were aged between 51 and 60 years, thus representing an older and more experienced sample. In terms of specialties, in Greece the largest proportion of the participants were pathologists (14.1%), followed by paediatricians (10.3%) and gynaecologists (9.1%), whilst in Cyprus the percentages were as following: paediatricians (17.1%), pathologists (9.6%), gynaecologists and surgeons (9.1%). A larger percentage of Cypriot participants (49.2% versus 26.7%) had a Master of Science (M.Sc) degree, whilst a larger proportion of Greek physicians (50.9% versus 30.6%) had participated in a publication during the previous five years. The great majority of doctors in both countries attend at least one conference per year. In terms of computer acquaintance the Cypriot physician cohort appears to have a slight advantage. However, it needs to be noted that physicians in both countries are more computer literate than the general population.

**Table 1:** Physicians characteristics in Greece and Cyprus

	**Greece** **(%)**	**Cyprus** **(%)**
**Sex**		

Male	79.9	69.8
Female	20.1	30.2
**Age**		

30–40	10.2	17.4
41–50	33.6	46.8
51–60	35.9	33.2
61+	20.3	2.6
**Practicing years**		

1–5	1.5	4.8
6–10	4.6	11.7
11–15	15.4	21.8
16–20	18.0	29.9
21+	60.5	38.8
**Specialty**		

Pathologist	14.1	9.6
Paediatrician	10.3	17.1
Gynaecologist	9.1	9.1
Cardiologist	8.6	7.5
Neurologist	7.3	-
Surgeon	6.9	9.1
Other	43.7	47.6
**Education/Training**		

Post graduate	26.7	49.2
Continuous education	50.1	56.5
At least one conference/year	88.8	77.7
At least one publication/5 years	50.9	30.6
**Employment Sector**		

Private	52.8	35.2
Public	15.7	64.2
Social insurance	2.6	-
Multiple	27.3	0.5
**Use of PC**		

High	23.1	19.9
Satisfactory	33.3	52.9
Moderate	26.5	19.4
None	17.1	7.9

### Influential factors

As mentioned earlier, the first part of the questionnaire was intended to investigate the criteria which physicians take into consideration when making prescribing decisions and their sources of information regarding advances in pharmaceuticals. As indicated in Table 2, clinical effectiveness is the most important factor, reaching 94.85% and 93.26% in Greece and Cyprus, respectively and there was no statistically significant difference between the two. However, in Greece the drug form, the recommended daily dose, and patient's own preferences appear to be more important in the selection of the therapy. With regard to the cost of drugs to patients, about sixty percent of physicians take it less or more seriously into consideration when prescribing and there are no major differences between the two countries. In particular, cost is important to 46.59% and 51.30% and highly important to 15.95% and 11.40%, of Greek and Cypriot physicians respectively. The same picture applies in relation to patient insurance coverage, where about 69.97% and 64.77% of physicians take it into account in Greece and Cyprus, respectively.

**Table 2:** Prescribing behavior of physicians

	Which constitutes for you the basic criterion for selecting a drug?			
	
	**Greece**	**Cyprus**	**Difference**	**p-value**
Proven clinical effectiveness	94.85%	93.26%	1.59%	0.392
Pharmaceutical delivery mode	38.21%	9.84%	28.37%	0.000
Recommended daily dose	35.55%	4.15%	31.40%	0.000
Cost to the patient	41.86%	8.29%	33.57%	0.000
Patient preference	11.05%	0.52%	10.53%	0.000
Other	2.49%	3.63%	-1.14%	0.406
	
	Which sources do you take into account in justifying your prescription choices?			
	
	**Greece**	**Cyprus**	**Difference**	**p-value**
Publications in medical journals	73.75%	58.55%	15.20%	0.000
Medical textbooks	60.71%	44.04%	16.67%	0.000
Medical congress announcements	70.27%	69.43%	0.84%	0.813
Pharmaceutical sales representatives	51.99%	61.14%	-9.15%	0.016
Medical libraries and internet data bases	29.49%	28.50%	0.99%	0.757
Others	3.41%	0.52%	2.89%	0.000
	
	For what reasons do you search further information from the above sources on your prescription choice?			
	
	**Greece**	**Cyprus**	**Difference**	**p-value**
Dose	43.02%	41.45%	1.57%	0.668
Adverse drug reactions	81.48%	77.20%	4.28%	0.182
Interactions with other substances	67.11%	63.21%	3.90%	0.292
Provision during pregnacy	53.99%	56.48%	-2.49%	0.514
Provision during breast feeding	50.91%	44.56%	6.35%	0.097
Liver-renal disorders	49.25%	55.96%	-6.71%	0.079
Chronic disease	43.44%	21.24%	22.20%	0.000
	
	How important is the cost of drug in your prescription selection?			
	
	**Greece**	**Cyprus**	**Difference**	**p-value**
Highly important	15.95%	11.40%	4.55%	0.066
Important	46.59%	51.30%	-4.71%	0.218
Not very important	25.83%	30.05%	-4.22%	0.225
Not at all important	11.63%	7.25%	4.38%	0.035
	
	To which extend does the existence of patient insurance coverage, influence your selection?			
	
	**Greece**	**Cyprus**	**Difference**	**p-value**
Very much	20.97%	14.51%	6.46%	0.022
Enough	31.11%	23.32%	7.79%	0.021
Rather influence me	17.89%	26.94%	-9.05%	0.000
Almost not all	17.14%	20.73%	-3.59%	0.239
Not at all	12.90%	14.51%	-1.61%	0.548

### Information sources

Physicians derive information to guide and justify their prescription choices mainly from medical journals, medical textbooks, proceedings of medical congresses, sales representatives and the internet. Nonetheless, there are some differences between the two countries. Specifically, Greek doctors rely more on scientific publications and medical textbooks and less on pharmaceutical representatives. Analytically, medical journals and textbooks are preferred by 73.75% and 60.71% of Greek doctors, respectively, compared to 58.55% and 44.04% of Cypriots. On the other hand, pharmaceutical representatives are preferred as an information source by 61.14% of Cypriot doctors compared to 51.99% of Greeks. Physicians are looking for information on the above sources mainly regarding the recommended dose of drugs, their potential side effects, their use during pregnancy and breast feeding and their use in the presence of chronic renal or liver disease. There are no significant differences between the two countries in relation to the type of enquired information.

### Generic drug prescribing

Table 3 contains information regarding the responses of physicians in relation to generic drug use. It is noteworthy that about half of doctors in Greece find generic drugs excellent or satisfactory in terms of efficacy, safety and effectiveness (51.25%, 54.98% and 52.41% respectively). However, only 25.20% of them prescribe generic drugs often or very often. On the other hand, Cypriots appear to have a more positive view regarding generic drugs. In particular, about 60.10%, 67.88% and 62.18% of physicians respectively find their efficacy, safety and effectiveness excellent or satisfactory. Finally, regarding the feasibility of prescribing based on drug substance (International Non-proprietary Name, INN) only, 37.62% and 15.21% of Greek physicians consider it rather feasible or very feasible, respectively, while the respective figures for Cypriot doctors are 39.38% and 17.62%.

**Table 3:** Attitudes of physicians towards generic prescribing

	What do you think about the quality of generic drugs in comparison to their branded ones?			
	
	**Greece**	**Cyprus**	**Difference**	**p-value**
Excellent	4.71%	3.11%	1.60%	0.240
Satistactory	46.54%	56.99%	-10.45%	0.006
Average	31.92%	32.64%	-0.72%	0.842
Rather bad	10.29%	5.18%	5.11%	0.005
Bad	6.54%	2.07%	4.47%	0.000
	
	What do you think about the safety of generics in comparison to their branded ones?			
	
	**Greece**	**Cyprus**	**Difference**	**p-value**
Excellent	5.31%	6.22%	-0.91%	0.614
Satistactory	49.57%	61.66%	-12.09%	0.001
Average	29.76%	26.42%	3.34%	0.326
Rather bad	8.99%	4.66%	4.33%	0.011
Bad	6.38%	1.04%	5.34%	0.000
	
	What do you think about the effectiveness of generics in comparison to their branded ones?			
	
	**Greece**	**Cyprus**	**Difference**	**p-value**
Excellent	5.79%	2.59%	3.20%	0.015
Satistactory	46.62%	59.59%	-12.97%	0.001
Average	33.49%	32.12%	1.37%	0.690
Rather bad	8.40%	3.11%	5.29%	0.000
Bad	5.69%	2.59%	3.10%	0.021
	
	How often do you prescribe a generic product instead of a branded one?			
	
	**Greece**	**Cyprus**	**Difference**	**p-value**
Very often	3.65%	13.99%	-10.34%	0.000
Often	21.55%	52.85%	-31.30%	0.000
Rarely	47.26%	27.98%	19.28%	0.000
Hardly ever	27.54%	5.18%	22.36%	0.000
	
	Do you think it is feasible to implement a prescribing system based on the INN?			
	
	**Greece**	**Cyprus**	**Difference**	**p-value**
Very feasible	15.21%	17.62%	-2.41%	0.397
Feasible	37.62%	39.38%	-1.76%	0.631
Rather impossible	30.60%	32.64%	-2.04%	0.567
Impossible	16.56%	10.36%	6.20%	0.011

### New drug prescribing

Table 4 presents information regarding attitudes in relation to new drugs. The majority, 82.11%, of physicians in Greece believe that a higher price does not necessarily imply better patient outcomes. This percentage is somewhat lower, 63.21%, in Cyprus and there are statistically significant differences between the two countries. There are also differences in relation to the perceptions of new drug effectiveness. More specifically, only 62.35% of Greek physicians believe that they are more effective, whilst the corresponding number amongst Cypriot doctors is 85.49%. Finally, regarding the sources of information about new product launches, these mainly include medical journals, congresses and sales representatives and secondarily scientific medical societies and the internet.

**Table 4:** Attitudes of physicians towards innovation

	In your opinion, does the high price of a new product implies also better effectiveness?			
	
	**Greece**	**Cyprus**	**Difference**	**p-value**
Absolutely	2.90%	4.15%	-1.25%	0.395
Enough	14.99%	32.64%	-17.65%	0.000
Not really	49.23%	55.96%	-6.73%	0.079
Almost not	10.48%	4.66%	5.82%	0.001
Not at all	22.40%	2.59%	19.81%	0.000
	
	Generally, what do you think about the effectiveness of new drug products in comparison to the older ones already on the market?			
	
	**Greece**	**Cyprus**	**Difference**	**p-value**
Clearly more effective	18.73%	24.35%	-5.62%	0.084
Rather more effective	43.62%	61.14%	-17.52%	0.000
Do not differ significantlly	30.15%	14.51%	15.64%	0.000
Do not differ at all	7.49%	0.00%	7.49%	0.000
	
	Which sources do you consult in order to get information for the launch of new drugs?			
	
	**Greece**	**Cyprus**	**Difference**	**p-value**
Scientific journals	78.99%	76.68%	2.31%	0.477
Medical congresses	75.10%	7.25%	63.85%	0.000
Pharmaceutical representatives	77.33%	88.08%	-10.75%	0.000
Scientific medical societies	38.87%	51.30%	-12.43%	0.001
Internet	30.32%	41.45%	-11.13%	0.003

### Adverse drug reactions and safety

Finally, Table 5 presents information regarding physician attitudes towards safety issues. As indicated by the finding, physicians get information about side effects primarily from the National Organisation for Medicines and secondarily from the internet, pharmaceutical companies, colleagues, and the media. In Cyprus the National Drug Agency and colleagues appear to have a less important role. Side effects appear in both countries to be a major cause of prescription choice modification, as more than 90% of doctors declare that they change their prescription patterns in cases of side effects. However, that 46.68% of doctors in Greece declared that they had not encountered any side effect during the two years prior to the study. It is also noteworthy that the majority of doctors do not inform the authorities about their own cases of side effects.

**Table 5:** Attitudes of physicians towards safety

	Which sources do you consult in order to get informed about the ADRs of a drug?			
	
	**Greece**	**Cyprus**	**Difference**	**p-value**
National Medicines Organization	65.70%	48.70%	17.00%	0.000
Internet	33.06%	37.82%	-4.76%	0.200
Pharmaceutical representatives	42.61%	41.97%	0.64%	0.866
Media	16.86%	11.92%	4.94%	0.052
Colleagues	38.54%	21.24%	17.30%	0.000
Other	4.32%	11.40%	7.08%	0.001
	
	The appearance of ADRs affects your prescribing decision?			
	
	**Greece**	**Cyprus**	**Difference**	**p-value**
Very much	51.21%	43.01%	8.20%	0.030
A lot	43.13%	47.15%	-4.02%	0.300
A little	4.91%	9.84%	-4.93%	0.026
Not at all	0.75%	0.00%	0.75%	0.000
	
	How many times have an ADR appeared in your patients in the past two years?			
	
	**Greece**	**Cyprus**	**Difference**	**p-value**
None	46.68%	8.81%	37.87%	0.000
1–5 times	46.26%	57.51%	-11.25%	0.004
6–10 times	5.04%	19.69%	-14.65%	0.000
More	2.02%	13.99%	-11.97%	0.000
	
	For such cases did you send a "yellow card*" to inform the authorities?			
	
	**Greece**	**Cyprus**	**Difference**	**p-value**
Yes	37.43%	11.92%	25.51%	0.000
No	62.57%	88.08%	-25.51%	0.000

*Yellow card is a form that needs to be filled by the physician if side effects appear in order to inform the authorities.

## Discussion

According to the present analysis, the Greek and Cypriot physicians are well educated and participate frequently in medical conferences. Furthermore, Greek doctors appear to have a high publication activity compared to their Cypriot colleagues. This could be partly explained by the fact that there are many university hospitals and also several medical journals in Greece. Our results indicate that there are similarities but also differences between physicians in Greece and Cyprus regarding the basic criteria upon which they select a pharmaceutical treatment. Particularly, the findings show that drug clinical effectiveness is the most important factor considered by physicians when they prescribe drugs. However, pharmaceutical form, recommended daily dose, and patient own preferences are taken into consideration more by Greek physicians compared to their Cypriots colleagues. Furthermore, doctors in both countries declare that they are sensitive to the economic burden imposed upon patients and the health system in general. It is notable that, although Cypriot physicians claim that the cost of the drug and insurance coverage are important factors during prescription, in fact they do not consider cost as one of the most important criteria for prescription choice.

The results of our study show that there are differences between the two countries regarding the sources of information. Specifically, Greek doctors claim to acquire more information from publications (journals, proceedings of conferences and textbooks) and less information from pharmaceutical sales representatives. The opposite happens in Cyprus, where proceedings of conferences and information from sales representatives are in the leading places, followed by publications. The classification of these information sources is different in the case of information regarding new drugs and adverse drug reactions. Specifically, in the case of new drugs, sales representatives are the first source of information in Cyprus and the second in Greece, right after scientific or medical journals. These data are consistent with those from other studies, where it has been also shown that pharmaceutical sales representatives are highly influential on decisions to prescribe new drugs [17,18].

Regarding opinions about generics and generic prescribing there are some differences between physicians in the two countries. In Greece doctors seem more cautious about the effectiveness, safety and efficacy of generic drugs, and as a result they do not prescribe generic drugs as a means to curtail expenditure. This is not so much the case in Cyprus, where doctors have a greater trust in generic drugs and prescribe them to a greater degree. However, in any case it needs to be stressed that, the percentage of physicians who believe that the quality of generics is excellent, is very small in both countries. This finding could indicate that physicians do not trust the process of approval and certification for generic products. The pessimistic and negative view in Greece needs further investigation in order to be addressed, as generic substitution is a major cost containment measure in most countries. Perhaps the low generic drug use in this country can be explained through the combination of several factors. As noted above a low generic quality perception prevails. This is combined with no major price differentials between branded and generic drugs, which eliminates the financial motive for the substitution. Furthermore, in Greece there are no financial incentives to motivate physicians to prescribe generics and to promote the generic market.

Finally, in the case of adverse drug reactions physicians are informed primarily by the National Organisation of Medicines and secondly by sales representatives. This is expected since the National Organisation of Medicines is responsible for the assessment of safety and the pharmacovigilance of medicinal products. Even though adverse drug reactions may not appear very often, they do have a profound effect on a physician prescribing patterns, so doctors seek information in order to be protected and prepared. It is notable, however, that when they encounter such problems physicians rarely inform the authorities accordingly, perhaps because they do not want to acknowledge the fact that their patients had side effects, or they do not evaluate the side effect as important. This finding is confirmed by data from the National Organisation for Medicines (EOF) in Greece. According to the Organisation, the reports about adverse drug reactions come mainly from pharmaceutical companies and from hospitals and to a much lesser degree from individual physicians [19]. For this reason, a campaign is under way in Greece aiming towards motivating more physicians to fill out the yellow card when they encounter an adverse drug reaction.

In conclusion, the findings of this study are in line to those of other published studies regarding physician prescribing behaviour and attitudes. More specifically, the issues of drug cost and patient insurance coverage concern the majority of physicians, who take these factors into consideration during their prescribing decisions. Results from a qualitative study in Denmark showed that drug price was considered an important factor influencing prescribing decision. Additionally, pharmaceutical industry sales representatives influenced physicians significantly [20]. Reichert at al showed in their study that physicians felt the cost of medicines to be an important criterion in prescription choice and this was even more important when patients were not insured [21]. In addition, our results are comparable to those of two other studies regarding opinions about generics and generic substitution [22,23]. Finally, a study that was carried out in 2005 in New Zealand, regarding the information sources which are mostly used by physicians, had similar results to our own. It showed that publications and pharmaceutical sales representatives were the most popular sources of information and that internet-based sources were used by half of the participants [24].

The present study is subject to all limitations that apply to surveys where postal questionnaires are used as a tool to extract data [25]. Nonetheless, the high response rate in both countries may allow us to believe that the study was successful. Another limitation is the lack of distinction between primary and secondary care physicians, which would have allowed a better comparison with other studies in this field. However, this parameter was not taken into account during the study design because, as mentioned at the beginning of this paper, Greece and Cyprus have no gate-keeping systems and patients have free access to every physician of every specialty and level of care.

## Conclusion

The present article provides valuable information regarding the prescribing behaviour of doctors in Greece and Cyprus. Further analysis will help us to understand further the association between prescription choices and physician characteristics. These insights will help policy makers in both countries to develop measures which could be used to achieve to greater clinical effectiveness and economic efficiency from drug prescribing.

## Competing interests

The authors declare that they have no competing interests.

## Authors' contributions

MT, VT, AP, JK and EP designed and coordinated the study in Greece and Cyprus, whilst VF and NM undertook the analysis of data and the writing of the manuscript. All authors read and approved the final manuscript.

## Pre-publication history

The pre-publication history for this paper can be accessed here:


